# Integrative Analysis of KCNK Genes and Establishment of a Specific Prognostic Signature for Breast Cancer

**DOI:** 10.3389/fcell.2022.839986

**Published:** 2022-05-17

**Authors:** Yutian Zou, Jindong Xie, Wenwen Tian, Linyu Wu, Yi Xie, Shanshan Huang, Yuhui Tang, Xinpei Deng, Hao Wu, Xinhua Xie

**Affiliations:** Department of Breast Oncology, Sun Yat-sen University Cancer Center, State Key Laboratory of Oncology in South China, Collaborative Innovation Center for Cancer Medicine, Guangzhou, China

**Keywords:** breast cancer, KCNK, biomarker, prognostic signature, tumor microenvironment

## Abstract

Two-pore domains potassium channel subunits, encoded by KCNK genes, play vital roles in breast cancer progression. However, the characteristics of most KCNK genes in breast cancer has yet to be clarified. In this study, we comprehensively analyzed the expression, alteration, prognosis, and biological functions of various KCNKs in breast cancer. The expression of KCNK1/4/6/9/10/13 were significantly upregulated, while KCNK2/3/5/7/17 were downregulated in breast cancer tissues compared to normal mammary tissues. Increased expression of KCNK1/3/4/9 was correlated with poor overall survival, while high expression of KCNK2/7/17 predicted better overall survival in breast cancer. Eight KCNK genes were altered in breast cancer patients with a genomic mutation rate ranged from 1.9% to 21%. KCNK1 and KCNK9 were the two most common mutations in breast cancer, occurred in 21% and 18% patients, respectively. Alteration of KCNK genes was associated with the worse clinical characteristics and higher TMB, MSI, and hypoxia score. Using machine learning method, a specific prognostic signature with seven KCNK genes was established, which manifested accuracy in predicting the prognosis of breast cancer in both training and validation cohorts. A nomogram with great predictive performance was afterwards constructed through incorporating KCNK-based risk score with clinical features. Furthermore, KCNKs were correlated with the activation of several tumor microenvironment cells, including T cells, mast cells, macrophages, and platelets. Presentation of antigen, stimulation of G protein signaling and toll-like receptor cascaded were regulated by KCNKs family. Taken together, KCNKs may regulate breast cancer progression via modulating immune response which can serve as ideal prognostic biomarkers for breast cancer patients. Our study provides novel insight for future studies evaluating their usefulness as therapeutic targets.

## Introduction

According to the estimates of global cancer statistics, breast cancer is known as the most common cancer and the second leading cause of cancer-related death among women (Kuang et al., 2015). In the United States, approximately 268,600 new cases and 42,260 deaths due to female breast cancer are expected to occur in the year 2019 (Heurteaux et al., 2004). Although surgery, radiation, and chemotherapy vastly improve the prognosis of patients with breast cancer, residual tumor cells may contribute to metastatic recurrence and death (Rolfsen et al., 2014). Potassium ion channels are the most widely distributed types in ion channels and are found in most cell types with multiple cellular functions. There are four major types of potassium channels: 1) Calcium-activated potassium (KCa) channels which turn on according to the presence of calcium ions or other signaling molecules. 2) Inwardly rectifying potassium (Kir) channels that pass current more easily in the inward direction. 3) Two-pore domain potassium (K2P) channels that are constitutively open or possess high basal activation. 4) Voltage-gated potassium (KV) channels, which can be switched on or off in response to changes in transmembrane voltage (Kuang et al., 2015).

As an important member of the potassium channel family, K2P channels (encoded by KCNK genes) have been studied and identified to be associated with a range of physiological and pathological processes, including neuroprotection, cardiac activity regulation, anesthesia, depression, and cancer (Heurteaux et al., 2004; Heurteaux et al., 2006; Lalevée et al., 2006; Devilliers et al., 2013; Innamaa et al., 2013). Moreover, the KCNK channels have been divided into six categories, including TWIK (tandem of P domains in weak inward rectifying K+ channels), TREK (TWIK-related K+ channels), TASK (TWIK-related acid-sensitive K+ channels), TALK (TWIK-related alkaline pH activated K+ channels), THIK (tandem-pore domain halothane inhibited K+ channels), and TRESK (TWIK-related spinal cord K+ channels) (Sauter et al., 2016).

The potential importance of KCNK genes in cancer has become of great interest in recent years. For example, KCNK2/10 were overexpressed in ovarian cancer, and KCNK2 regulators played an important role in cell proliferation and apoptosis. TREK-1(KCNK2) may be a promising novel target for pancreatic ductal adenocarcinoma (Sauter et al., 2016). The TREK2 channel was present in bladder cancer cell lines and may contribute to cell cycle-dependent growth (Park et al., 2013). TASK-1 Regulates Apoptosis and Proliferation in a Subset of Non-Small Cell Lung Cancers (Leithner et al., 2016). TASK3 was significantly up-regulated whereas TASK1 and TRESK were both significantly down-regulated in advanced, poorly differentiated oral squamous cell carcinoma (Zavala et al., 2019). Overexpression rather than mutation of KCNK9 may contribute to the development of colorectal cancer (Kim et al., 2004). Knockdown of TREK-1 significantly inhibited the proliferation of prostate cancer cells both *in vitro* and *in vivo* (Zhang et al., 2015). Due to the increase in apoptosis, knocking down of TASK-3 reduced cell proliferation and vitality (Cikutović-Molina et al., 2019). Lower expression of KCNK 2/15/17 in liver cancer and overexpression of KCNK9 were associated with better prognosis in hepatocellular carcinoma (Li et al., 2019).

Several studies have focused on the role of KCNK genes in tumor treatment and prevention strategies for breast cancer (Wallace et al., 2011). E2 induced the overexpression of KCNK5 in luminal breast cancer cells through ER*α*+, and KCNK5 played an important role in regulating cell proliferation (Alvarez-Baron et al., 2011). Increased TASK-3 expression, which could be modulated by PKC activation, reduced cell migration and invasion in triple-negative breast cancer cells (Lee et al., 2012). Overexpression of KCNK5, KCNK9, and KCNK12, as well as reduced expression of KCNK6 and KCNK15, was significantly correlated with triple-negative subtype in breast carcinoma (Dookeran et al., 2017). In this study, we comprehensively analyzed the expression, alteration, prognosis, and biological functions of various KCNKs in breast cancer. A specific prognostic signature with seven KCNK genes was established using machine learning method. This constructed model manifested accuracy in predicting the prognosis of patients with breast cancer.

## Materials and Methods

### Data Collection

The 15 members of the KCNK gene family were acquired from reported literature. The mRNA expression matrix of KCNK genes and corresponding clinical data of breast cancer patients were downloaded from the TCGA database (https://portal.gdc. cancer. gov/). Only samples with complete prognostic data were included.

### General Assessment and Visualization of KCNK Genes

To investigate the clinical correlation of KCNK genes, boxplots and heatmaps were used to display the distribution of KCNK gene expressions and their relationship with various clinic features in breast cancer patients. Wilcoxon test was applied to calculate statistical significances between groups. The position and expression levels of 15 KCNK genes were shown with a circos plot by applying the “RCircos” R package (Zhang et al., 2013). The correlation of each KCNK gene was shown utilizing the “corrplot” R package. Genomic alteration landscape and biological function enrichment of KCNK gene family in breast cancer in breast cancer was explored in the cBioPortal website (http://www.cbioportal.org/).

### Survival Analysis

To better evaluate the prognostic value of the differentially expressed KCNK genes, we obtained the best cutoff value by the “survminer” R package. all samples were divided into high and low expression groups with the automatically selecting cutoff. Kaplan-Meier analysis was performed with the “survival” R package.

### Construction and Validation of the KCNK Gene Signature

7 KCNK genes, including KCNK1, KCNK2, KCNK3, KCNK4, KCNK7, KCNK9, and KCNK17, were included to construct a risk signature for their significant effects on overall survival (OS) in breast cancer patients. The “sample” function in R was used and the TCGA dataset was then randomly separated into a training dataset and a validation dataset with the split ratio of 55% and 45%. Based on the LASSO regression analysis, we determined the coefficients and calculated the risk score of each patient with the formula as follows:

$$Risk\;score=\sum_{i=1}^{7}Coefi\;*\;Expi$$ (1)

Coefi denote the risk coefficient and Expi refer to the expression of each gene, respectively. To make plots more intuitionistic, we used a linear transformation to adjust the risk score. The calculated risk score minus the minimum and divided it by the maximum, which mapped these exponentials to the range of 0–1 (Xie et al., 2021).

According to the median value of the risk score, patients were divided into high- and low-risk groups, and the OS of the two subgroups were compared by Kaplan-Meier analysis with the log-rank test. Similarly, these analyses described above were also conducted in the validation set.

### Establishment of a Predictive Nomogram

Univariate and multivariate cox regression analyses were utilized to identify independently prognostic factors in breast cancer patients of the training set. The nomogram was constructed and shown with the “regplot” R package. Then, calibration plots were plotted to evaluate the reliability of the nomogram, and the decision curve analysis (DCA) was performed to investigate the clinical net benefit of the nomogram (R package “caret” and “rmda”).

### Estimation of Immune Microenvironment

Tumor Immune Estimation Resource (TIMER) (Li et al., 2020) and CIBERSORT (Newman et al., 2015) algorithms were applied to compare the infiltration levels of various immune cells between KCNKs, as well as the high-risk and low-risk groups.

### Construction of PPI Networks

The protein-protein interaction (PPI) network of every candidate KCNK gene was built based on the STRING database (Szklarczyk et al., 2015) and rebuilt with Cytoscape software (Shannon et al., 2003).

### UALCAN Analysis

UALCAN (http://ualcan.path.uab.edu) is an online tool for in-depth analysis of gene expression differences between various cancers and normal samples (Chandrashekar et al., 2017). In this study, we reviewed the relationship between the expression levels of each model gene and the breast cancer subtypes, histological subtypes, and pathologic stages.

### Collection of Clinical Samples

Fresh breast cancer tissues and adjacent mammary tissues were collected from breast cancer patients who received surgery in Sun Yat-sen University Cancer Center (SYSUCC). All resected samples were immediately stored in RNAlater (Ambion, TX). This study was approved by the Ethics Committee of SYSUCC. Written informed consent was collected from all patients.

### Cell Lines and Cell Culture

Human breast cancer cell lines were purchased from the American Type Culture Collection. All cell lines were cultured following standard guidelines. All cell lines were maintained without antibiotics in an atmosphere of 5% CO_2_ and 99% relative humidity at 37°C. Cell lines were passaged for fewer than 6 months and were authenticated by short tandem repeat analysis. No *mycoplasma* infection was found for all cell lines.

### RNA Isolation and Quantitative Real-Time PCR Analysis

Total RNA of cells was extracted with RNA-Quick Purification Kit (ES-RN001, Shanghai Yishan Biotechnology Co.). The primer sequences are provided in Supplementary Table S1. The quantitative real-time PCR (qRT-PCR) plate was employed from NEST NO.402301. RNA levels were determined by qRT-PCR in triplicate on a Bio-Rad CFX96 using the SYBR Green method (RR420A, Takara). The RNA levels were normalized against *β*-actin RNA using the comparative Ct method.

## Results

### Aberrant Expression of KCNK Gene Family in Breast Cancer

**FIGURE 1:**
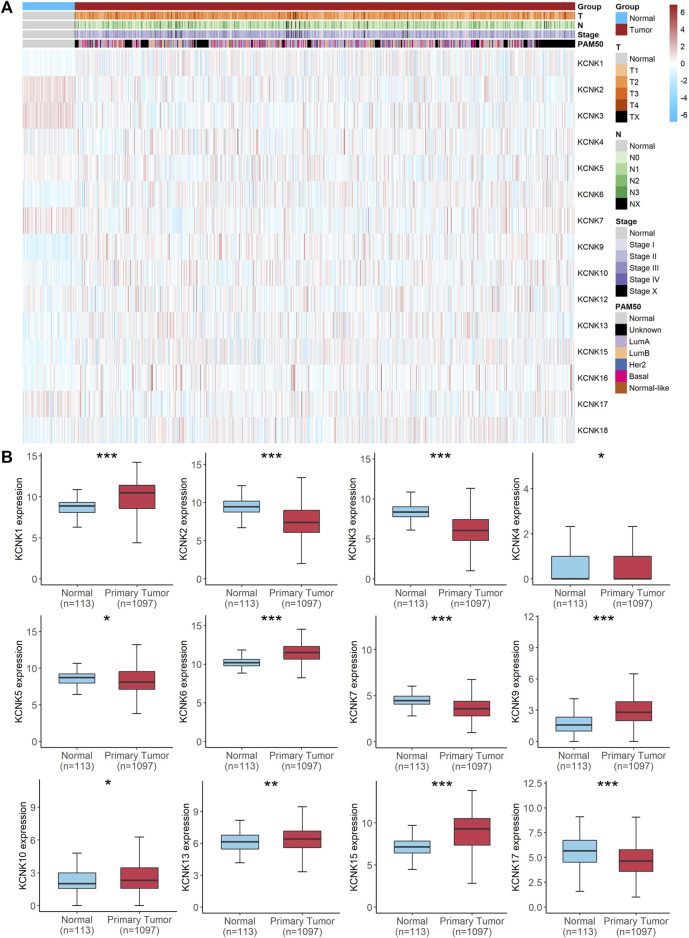
Aberrant expression of KCNK gene family in breast cancer. **(A)** Heatmap of the expression of 15 KCNKs and corresponding clinical information of patients with breast cancer according to the TCGA database. **(B)** Boxplots of the differently expressed KCNKs in normal mammary and breast cancer tissues.

To understand the mRNA expression of KCNK genes in breast carcinoma samples, we reviewed KCNK mRNA levels of 1,097 tumors and 113 normal tissues in the TCGA database. Totally, fifteen genes in the KCNK family were analyzed (KCNK1, KCNK2, KCNK3, KCNK4, KCNK5, KCNK6, KCNK7, KCNK9, KCNK10, KCNK12, KCNK13, KCNK15, KCNK16, KCNK17, KCNK18). Heatmap depicts the expression of different KCNK genes and clinical information in each breast cancer patient (Figure 1A). We found that twelve genes in the KCNK family were differentially expressed in normal and breast cancer tissues. Among them, KCNK1/4/6/9/10/13/15 were upregulated in breast tumor samples compared to normal mammary tissues. Additionally, KCNK2/3/5/7/17 were significantly downregulated in breast cancer samples (Figure 1B). The RNA expression level of KCNK2, KCNK5, KCNK9, KCNK13, and KCNK15 were validated in human breast cancer cell lines (Supplementary Figure S1). Our results revealed that KCNK9, KCNK13, and KCNK15 was significantly upregulated in breast cancer cell lines including MDA-MB-231 and SK-BR-3 cell lines, while KCNK2 and KCNK5 was downregulated in breast cancer cell lines comparing with breast epithelial cell line MCF-10A (Supplementary Figure S1A). To further validate their expression patterns in clinical specimens, we detected the expression level in breast cancer tissues and adjacent normal tissues. We found that KCNK2 and KCNK5 was downregulated in breast cancer, while KCNK9, KCNK13, and KCNK15 was upregulated in breast cancer (Supplementary Figure S1B). In addition, we found that breast cancer patients with metastasis disease have low expression of KCNK2 and KCNK13 (Supplementary Figure S1C).

### Genomic Alteration Landscape of KCNK Gene Family in Breast Cancer

**FIGURE 2:**
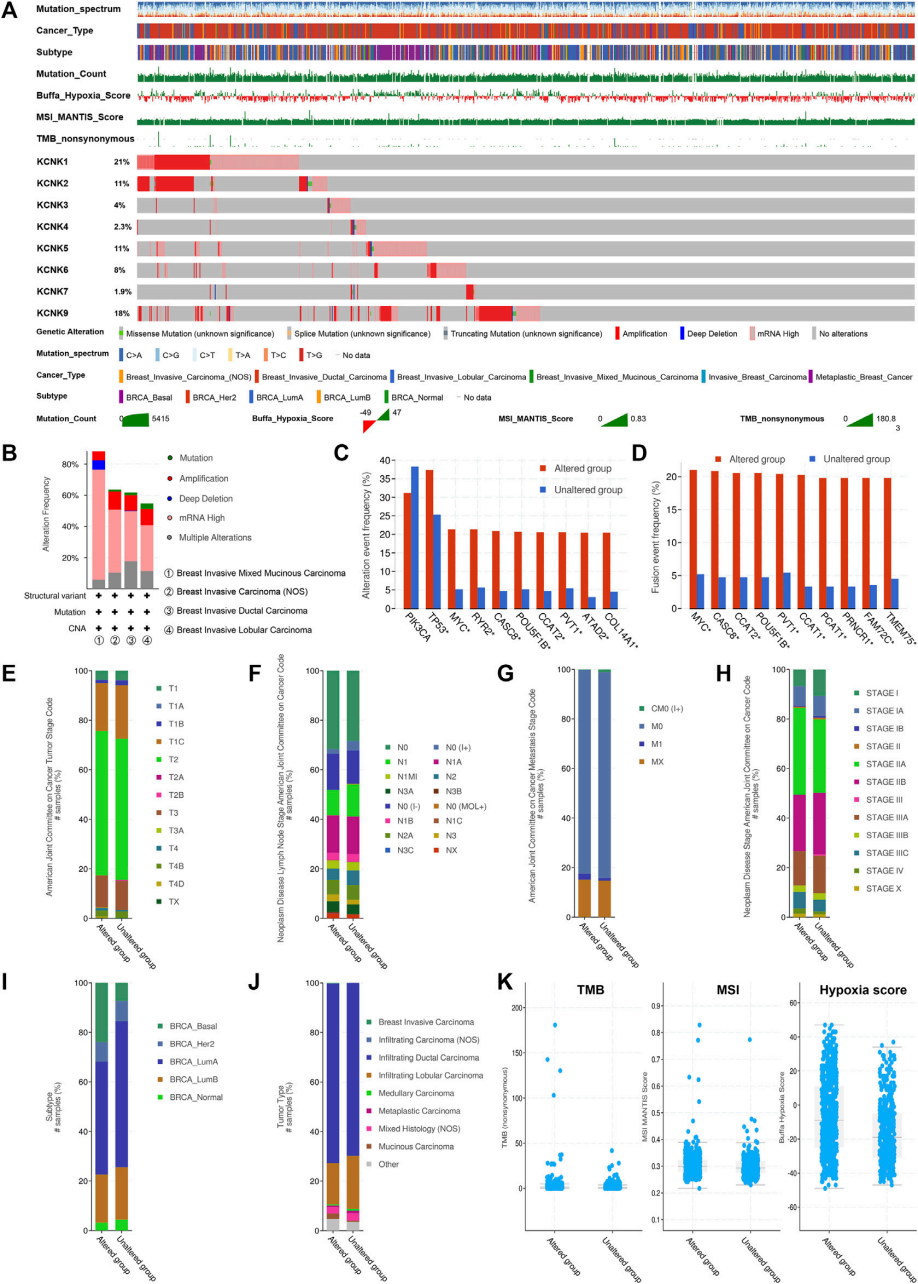
Genomic alteration landscape of KCNK gene family in breast cancer. **(A)** The genomic alteration of KCNK genes in TCGA cohort. Oncoplot was used for display. **(B)** The alteration frequency of KCNKs in different breast cancer pathology types. **(C)** The correlation between the alteration of KCNKs and the alteration of several robust oncogenes in breast cancer development. **(D)** The correlation between KCNKs and fusion genes. **(E–J)** The relationship between the alteration of KCNK genes and the clinical characteristics of breast cancer. **(K)** The relationship between the alteration of KCNK genes and tumor mutation burden (TMB), microsatellite instability (MSI), and hypoxia score in breast cancer.

We next evaluated the genomic mutations of KCNKs and their correlation with other clinical characteristics in breast cancer. A total of 1,082 patients were included for analysis on the TCGA PanCancer accessed by the cBioPortal tool (Figure 2A). The genomic mutation rate ranged from 1.9% to 21% among four different breast cancer subtypes. We found that KCNK1 and KCNK9 were the two most common mutations in breast cancer, occurring in 21% and 18% of patients, respectively (Figure 2A). The alteration frequency of KCNKs in different breast cancer pathology types was analyzed, in which breast invasive mixed mucinous carcinoma had the highest mutation rate of KCNK family genes (Figure 2B). Moreover, the rate of KCNK gene alteration was significantly correlated with the alteration of several robust oncogenes in breast cancer development (PIK3CA, TP53, MYC, PVT1, etc.) (Figure 2C). The correlation between KCNKs and fusion genes was further analyzed. We found that the frequency of several fusion genes was higher in the altered group of KCNK genes (Figure 2D). In addition, mutation of KCNK genes was associated with the clinical characteristics (T status, N status, M status, clinical stage, molecular subtype, and pathological type) in breast cancer (Figures 2E–J). Patients with high TMB, MSI score or hypoxia score had an increased rate of KCNK alteration (Figure 2K).

### Biological Function Enrichment of KCNK Gene Family in Breast Cancer

**FIGURE 3:**
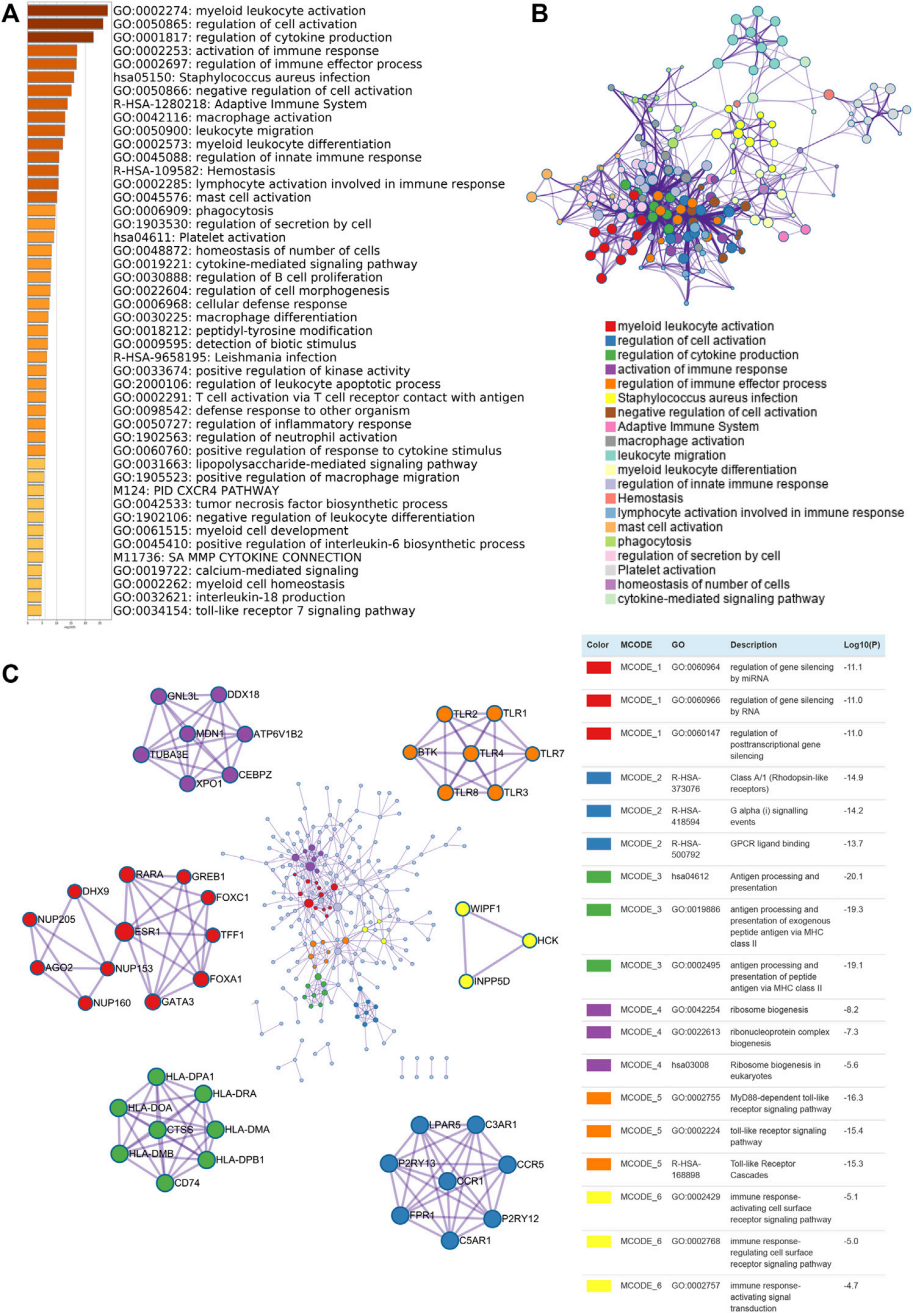
Biological function enrichment of KCNK gene family in breast cancer. **(A)** Heatmap of GO enriched terms colored by *p*-values. **(B)** Network of GO function and pathway enrichment analysis of KCNKs which was colored by cluster. **(C)** Protein-protein interaction (PPI) enrichment network analysis of KCNK related genes in breast cancer.

To investigate the potential biological function of KCNKs in breast cancer development and progression, we conducted function and pathway enrichment analysis. Genes correlated with KCNKs were identified by Spearman’s test (|r|>0.3, *p* < 0.05), which were included for further functional and pathway enrichment analysis in Metascape. We found that KCNKs were mainly associated with the regulation of tumor immune response (Figure 3A). KCNKs were involved in the activation of several tumor microenvironment components, including T cells, mast cells, macrophages, and platelets. Pathway enrichment analysis identified that KCNKs family might modulate immune response via regulating presentation of antigen, stimulation of G protein signaling, and toll-like receptor cascaded (Figure 3B). Furthermore, protein-protein interaction enrichment analysis was performed to figure out the hub genes of each biological function module (Figure 3C).

### Prognostic Value of the Expression of KCNK Gene Family in Breast Cancer

**FIGURE 4:**
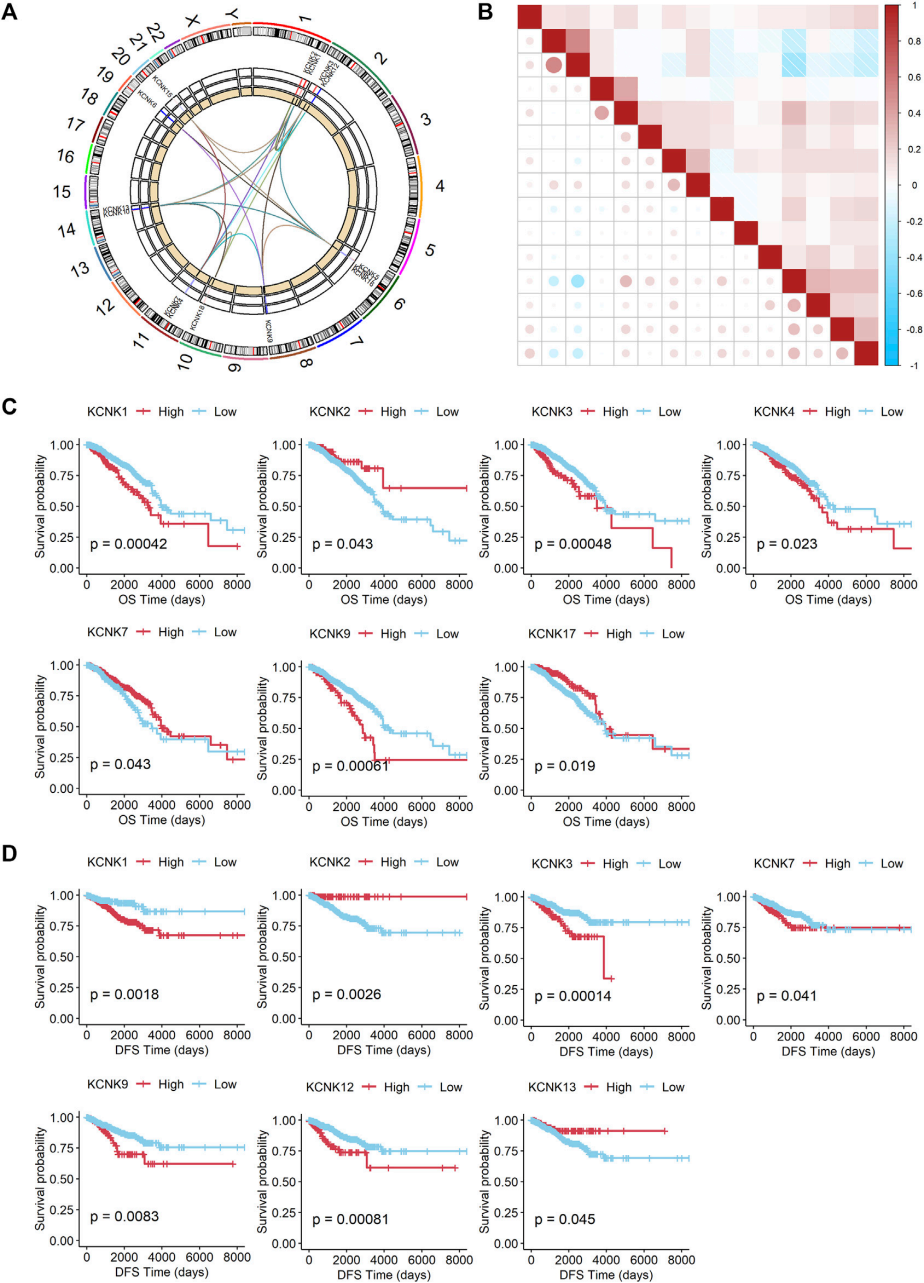
Prognostic value of the expression of KCNK gene family in breast cancer. **(A)** Circos plot displaying the position of KCNK genes on chromosomes. **(B)** The correlation of each KCNK gene according to the TCGA database. **(C)** K-M analysis of the overall survival (OS) of breast cancer patients with high or low expression of each KCNK gene according to the TCGA database. **(D)** K-M analysis of the disease-free survival (DFS) of breast cancer patients with high or low expression of each KCNK gene according to the TCGA database.

Firstly, the position and expression of KCNK genes were analyzed (Figure 4A), and the correlation of KCNK genes is also shown in Figure 4B. We next explored the prognostic predictive value of KCNK genes for patients with breast cancer by using the TCGA database. High expression of KCNK1/3/4/9 was correlated with a poor overall survival in patients with breast cancer (Figure 4C). Patients with higher KCNK2/7/17 expression were associated with better overall survival (Figure 4C). In addition, we found that KCNK1/3/7/9/12 was negatively correlated with disease-free survival in patients with breast cancer (Figure 4D). High expression of KCNK2 and KCNK13 levels were associated with favorable disease-free survival in patients with breast cancer (Figure 4D).

### Construction and Validation of a KCNK-Based Prognostic Signature for Breast Cancer

**FIGURE 5:**
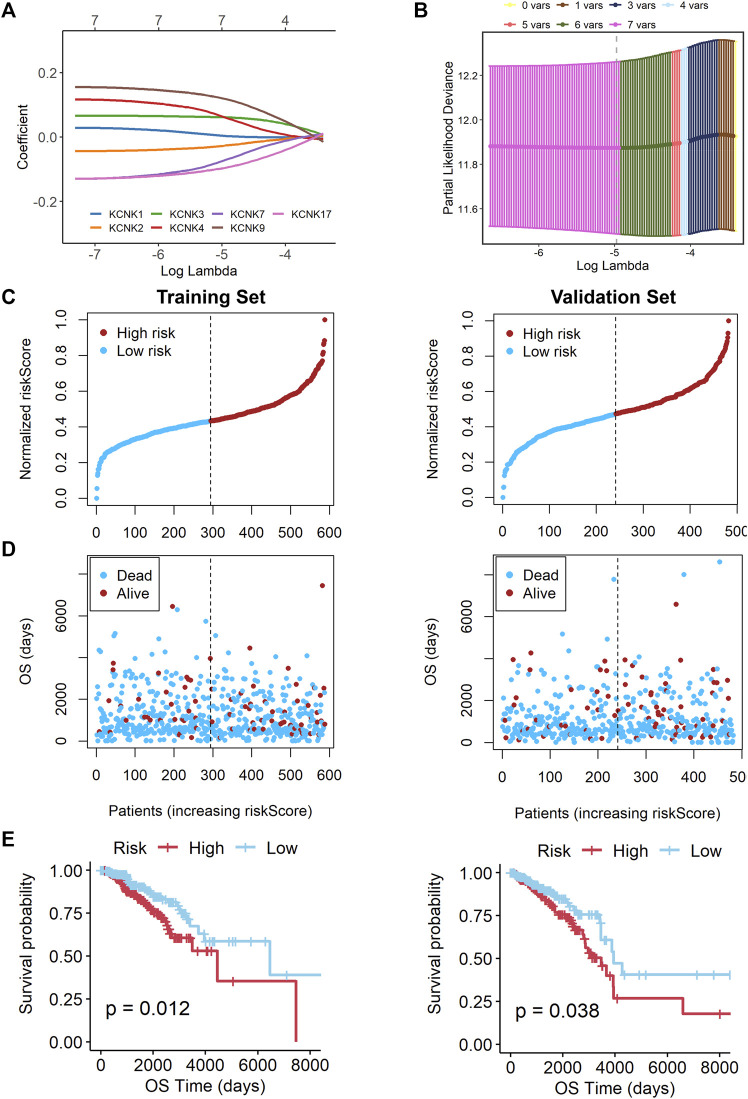
Construction of a KCNK-based prognostic signature for breast cancer. **(A)** Selection of the seven model KCNK genes in lasso regression model. **(B)** Cross-validation of the constructed signature. **(C,D)** Distribution of adjusted risk score in the training and validation cohorts. **(E)** Overall survival (OS) in the low- and high-risk group patients in the training and validation cohorts. The specific prognostic signature with seven KCNK genes was established, which manifested accuracy in predicting the prognosis of breast cancer in both training and validation cohorts.

A 7-gene signature was constructed by performing the LASSO Cox regression analysis (Figures 5A,B). The risk score was calculated by the formula as follows. risk score = (0.002199218 * KCNK1 exp.) + (−0.027946148 * KCNK2 exp.) + (0.060471745 * KCNK3 exp.) + (0.068412510 * KCNK4 exp.) + (−0.077890308 * KCNK7 exp.) + (0.120687908 * KCNK9 exp.) + (-0.096099063 * KCNK17 exp.).

Based on the median risk score, 588 patients in the training cohort were classified into low- and high-risk groups. We found that patients in the high-risk group were harder to survive as risk scores increased (Figures 5C,D). An obvious difference was detected in OS time between these two groups, that is, patients in the low-risk group were more likely to lower death rate (*p* = 0.012, Figure 5E). Subsequently, we utilized the validation cohort. Based on the median risk score, 482 patients were also divided into two groups. It also showed that high risk score resulted in poor survival time (Figures 5C,D). K-M analysis also showed that patients in the high-risk group were more likely to have shorter OS time and higher death rate (*p* = 0.038, Figure 5E).

### Establishment and Assessment of the KCNK-Based Nomogram Survival Model for Breast Cancer

**FIGURE 6:**
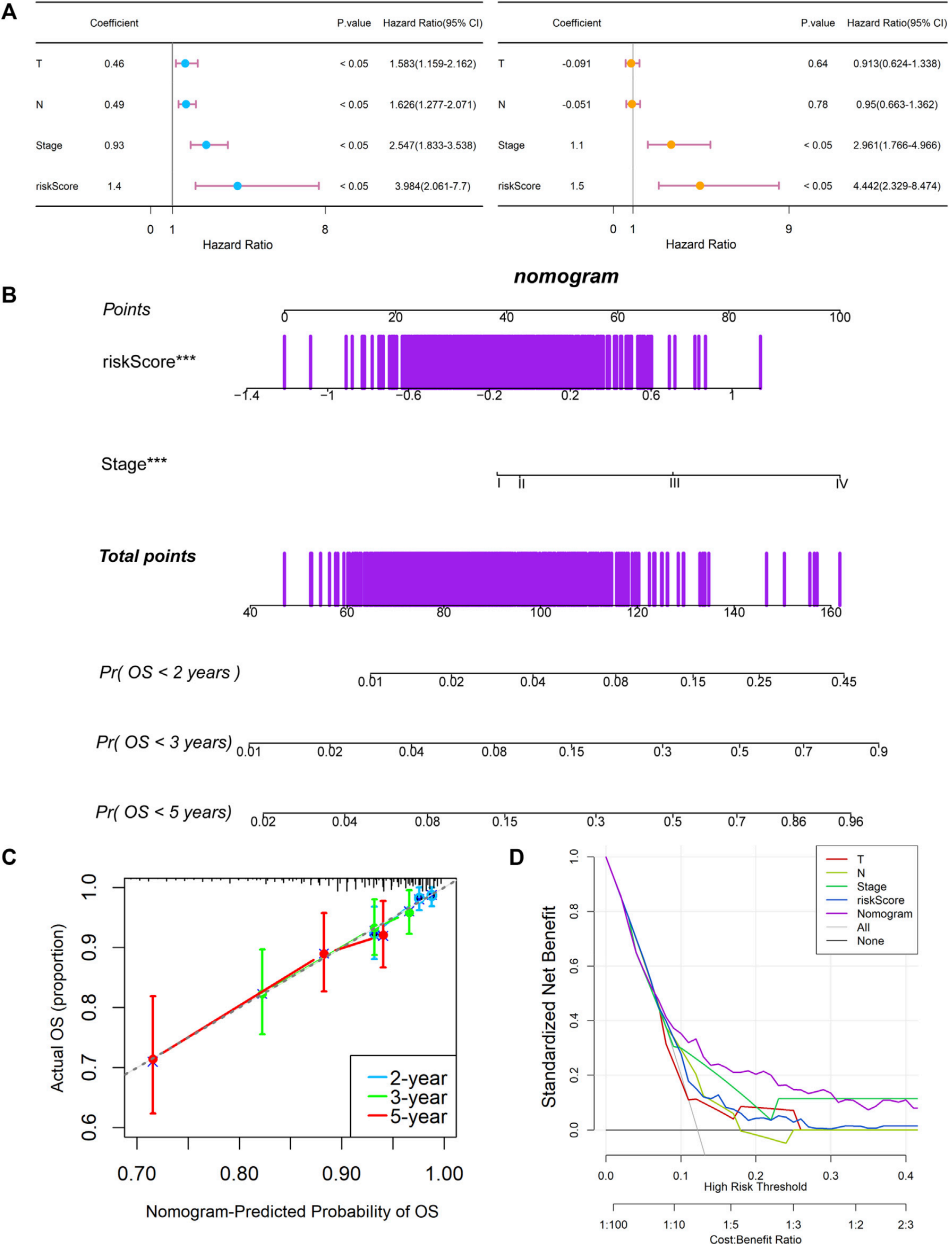
Establishment and assessment of the KCNK-based nomogram survival model for breast cancer. **(A)** Univariate and multivariate analysis for the training cohort. **(B)** The nomogram for predicting the overall survival of the patients with breast cancer. **(C)** Calibration plots showed the probability of 2-, 3-, and 5-years overall survival in training cohort. **(D)** DCA of nomogram predicting 2-, 3-, and 5-years overall survival.

Univariate and multivariable Cox regression analyses were performed to explore whether the risk score could be an independent prognostic factor. The univariate Cox regression analysis showed that, compared with other features, the risk score was obviously regarded as a risk factor (HR = 3.984, 95% CI: 2.061–7.700, and *p* < 0.05, Figure 6A). After adjusting for other confounding factors, the multivariate analysis also indicated that the risk score was still an independent prognostic factor (HR = 4.442, 95% CI: 2.329–8.474, and *p* < 0.05, Figure 6A). Besides, the relationship between the expression levels of each model gene and the breast cancer subtypes, histological subtypes, and pathologic stages were also analyzed in Supplementary Figure S1. Stage and risk score were selected to establish a KCNK-based nomogram model in the training cohort (Figure 6B). Calibration curves showed the accuracy of this model in predicting the 2-, 3-, and 5-years survival rate is favorable (Figure 6C). Moreover, we performed DCA and found that the nomogram model was apparently better than any other predictor applied in this study (Figure 6D).

### Relationship Between KCNK-Based Risk Model and Tumor Microenvironment Components in Breast Cancer

**FIGURE 7:**
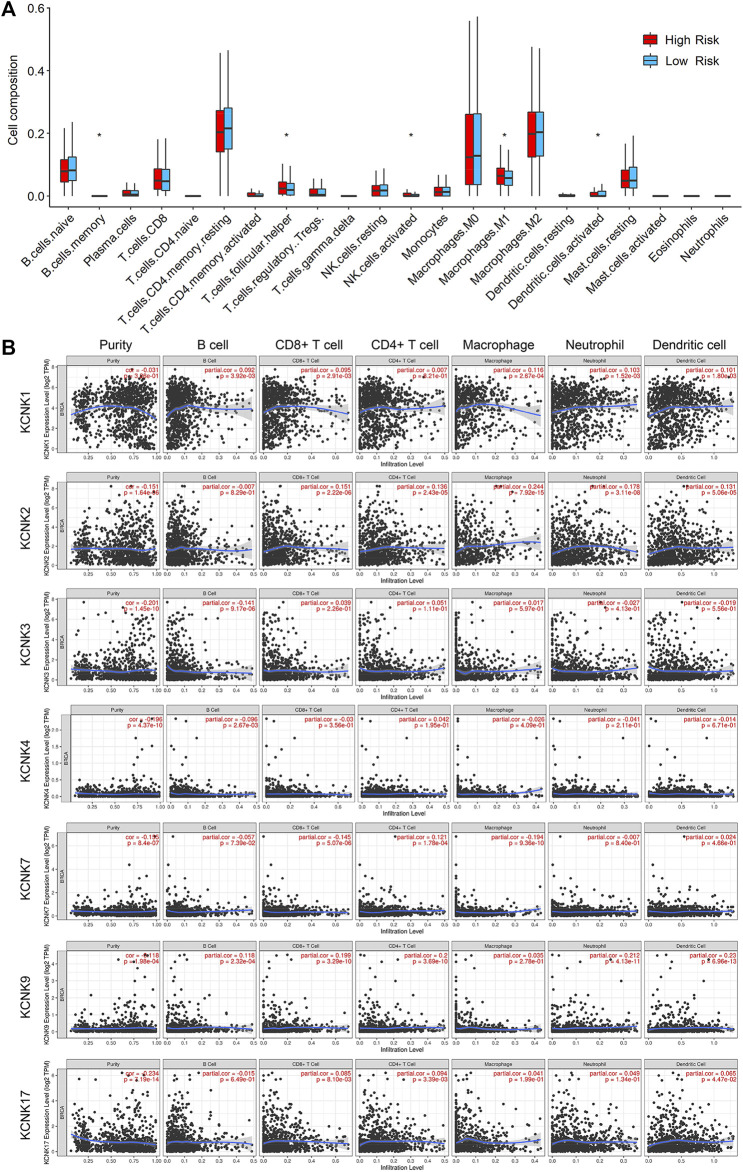
Relationship between KCNK-based risk model and tumor microenvironment components in breast cancer. **(A)** Boxplots of the infiltrated level of immune or stromal cells in the tumor microenvironment between high- and low-risk groups according to the TCGA cohorts. **(B)** The correlation between seven modeling KCNK genes and the infiltrated level of immune or stromal cells in the tumor microenvironment.

To validate the function of KCNKs in regulating the tumor microenvironment, we compared the infiltrated level of several immune and stromal cells between high and low-risk groups by using the CIBERSORT algorithm. We found that the KCCK-based risk score was correlated with the proportion of several tumor microenvironment components, including memory B cell, helper T cell, activated NK cell, M1 macrophage, and activated dendritic cell (Figure 7A). Moreover, the correlation between seven modeling genes and tumor microenvironment cells was analyzed. High expression of KCNK1 and KCNK2 was associated with increasing macrophage in breast cancer. Expression of KCNK7 was negatively correlated with CD8^+^ T cell level, while KCNK9 expression was positively correlated with CD8^+^ T cell level in breast cancer (Figure 7B). Furthermore, the relationship between copy number variation of KCNKs and tumor microenvironment components was also analyzed (Supplementary Figure S2).

## Discussion

The two-pore domain (K2P) potassium channel family encoded by KCNK genes has been reported to be involved in the development of breast cancer (Kindler and Yost, 2005). Our study was the first systematic and comprehensive analysis of all fifteen KCNK genes expression, alteration, prognostic value, and their potential biological functions in breast cancer. Through bioinformatics analysis, we found that twelve KCNKs were differentially expressed between normal mammary and breast cancer tissues. Moreover, eight mutations of KCNK genes were identified which were associated with more advanced clinical characteristics according to the TCGA database. Expression of KCNK1/2/3/4/7/9/17 and KCNK1/2/3/7/9/12/13 were the prognostic factor for overall survival and disease-free survival in patients with breast cancer, respectively. Though function enrichment analysis, we found that KCNKs were mainly associated with the regulation of tumor immune response. The immune system can recognize and destroy tumor cells, preventing them from growing and spreading (da Silva et al., 2013). Several tumor microenvironment cells were activated by KCNKs, including T cells, mast cells, macrophages, and platelets. Presentation of antigen, stimulation of G protein signaling, and toll-like receptor cascaded were regulated by KCNKs family. A specific prognostic signature with seven KCNK genes was established using machine learning method. This constructed model manifested accuracy in predicting the prognosis of breast cancer in both training and validation cohorts. Afterwards, a nomogram with great predictive performance was constructed through incorporating KCNK-based risk score with clinical features. In consist with the functional enrichment analysis, the established risk score was correlated with the infiltrated level of several immune and stromal cells in the tumor microenvironment.

Several KCNK genes were included in the specific prognostic model, which have been reported as important oncogenes or tumor suppressors in multiple malignancies. KCNK1, also called TWIK-1, was cloned from the human kidney and was the first K2P channel identified (Zhang et al., 2015). In our study, mRNA expression level of KCNK1 was upregulated in breast cancer tissues with poor overall survival and disease-free survival. However, further research was necessary to test whether KCNK1 could be a targeted signature in breast cancer therapy. KCNK2, also known as TREK-1, expressed highly in central nervous system and was found to be responsible for temperature, mechanical stretch (Bockenhauer et al., 2001). KCNK2 was detected as a prognostic factor in breast cancer, which was similar with previous study (Li et al., 2019). The KCNK9 gene, commonly referred to as TASK-3, was associated with cancer due to its overexpression in human tumors and its ability to promote tumor survival and growth (Zavala et al., 2019). Base on the previous research, KCNK9 was overexpressed in rectal cancer, melanoma, and adrenal cortical adenocarcinoma (Sun et al., 2016). KCNK9 has been identified as an oncogene to promote cell migration and invasion in breast cancer cells, which was consistent with our result that increased KCNK9 mRNA expression had worse disease-free survival (Rusznák et al., 2008). Besides, several previous studies regarding the impact of KCNKs in breast cancer focused on certain subtypes such as luminal and triple-negative subtype, which are different from all-subtype breast cancer studies.

Taken together, we determined KCNKs as ideal prognostic biomarkers for patients with breast cancer. Our results also provided more evidence for discovering potential therapeutic targets for breast cancer patients.

## Data Availability

The datasets presented in this study can be found in online repositories. The names of the repository/repositories and accession number(s) can be found in the article/Supplementary Material.
