# Supplementary material for: Integrative Analysis of KCNK Genes and Establishment of a Specific Prognostic Signature for Breast Cancer
Source: Front Cell Dev Biol. 2022 May 17;10:839986. doi: 10.3389/fcell.2022.839986 (PMC9152175; doi:10.3389/fcell.2022.839986)
Supplement: Supplementary file 1 [file DataSheet2.PDF]

Table S1. The primer sequences of KCNK2, KCNK5, KCNK9, KCNK13, KCNK15, and β-actin.

Gene Symbol	Primer sequence (5'-3')
KCNK2	F:ATAGTGGCAGCAATAAATGCAGG<br>R:TTCTGTGCGTGGTGAGATGTT
KCNK5	F:ACTGGCCCAATGCAATGATTT<br>R:CTGATCCACGTCAGGCAGAG
KCNK9	F:ACACTGACGTGTCTATGGAGA<br>R:GGTAGTCAACGTGATGAAGCAG
KCNK13	F:TCTGCTGCATCTACTCCTTGT<br>R:TTCCTGCGTGATCGCAAGAG
KCNK15	F:CTACGAGCACTGGACCTTCTT<br>R:CGTAAGGATGTAGACGAAGCTGA
β-actin	F:CATGTACGTTGCTATCCAGGC<br>R:CTCCTTAATGTCACGCACGAT
