# Supplementary material for: Integrative Analysis of KCNK Genes and Establishment of a Specific Prognostic Signature for Breast Cancer
Source: Front Cell Dev Biol. 2022 May 17;10:839986. doi: 10.3389/fcell.2022.839986 (PMC9152175; doi:10.3389/fcell.2022.839986)

**Supplement Figures**

**
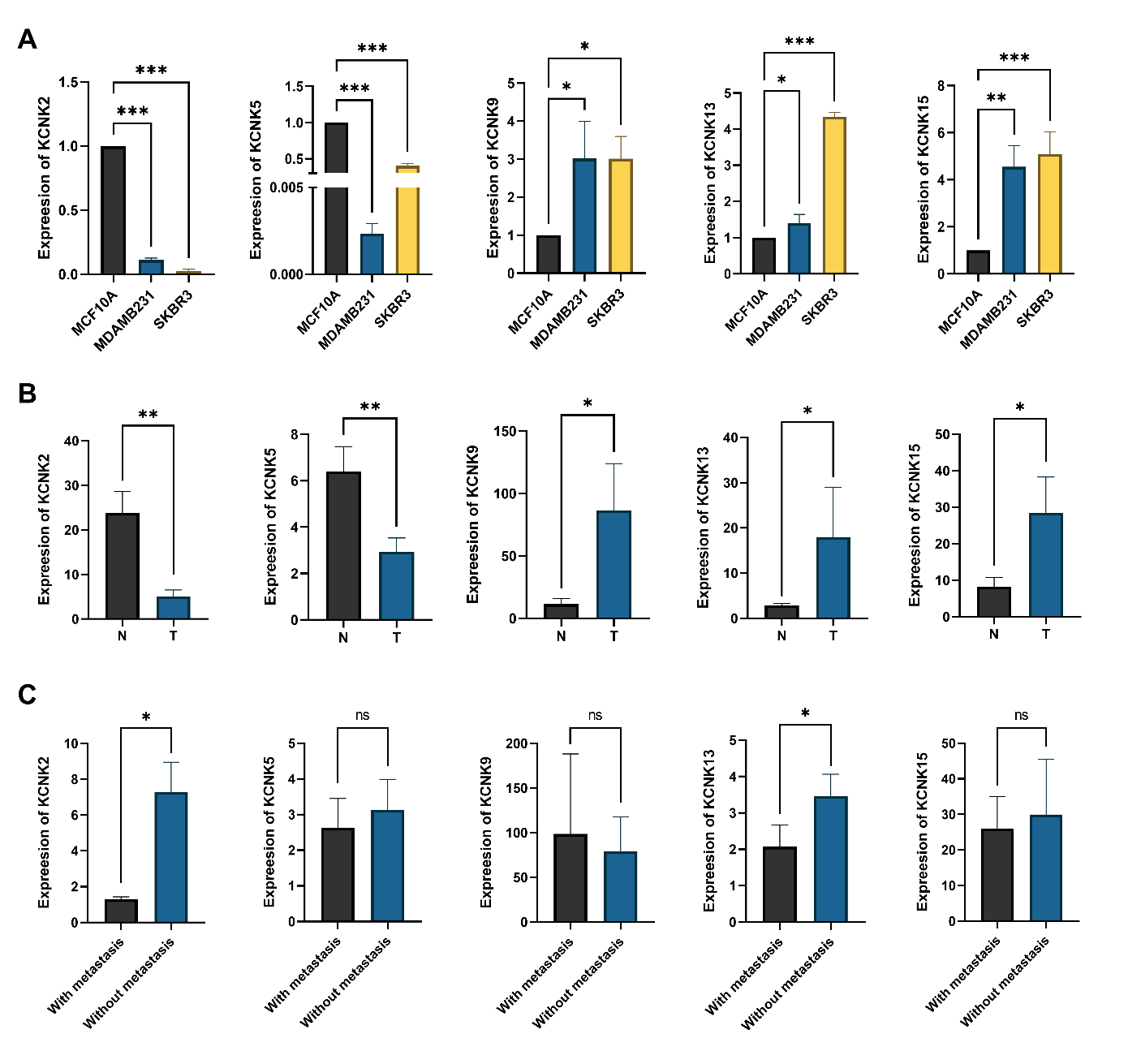
Supplement Figure S1.** (A) Relative expression level of KCNK2, KCNK5, KCNK9, KCNK13, and KCNK15 in human breast epithelial cell line (MCF-10A) and human breast cancer cell lines (MDA-MB-231 and SK-BR-3). (B) Relative expression level of KCNK2, KCNK5, KCNK9, KCNK13, and KCNK15 in breast cancer tissues and adjacent normal mammary tissues. (C) Relative expression level of KCNK2, KCNK5, KCNK9, KCNK13, and KCNK15 in breast cancer tissues with or without metastasis.


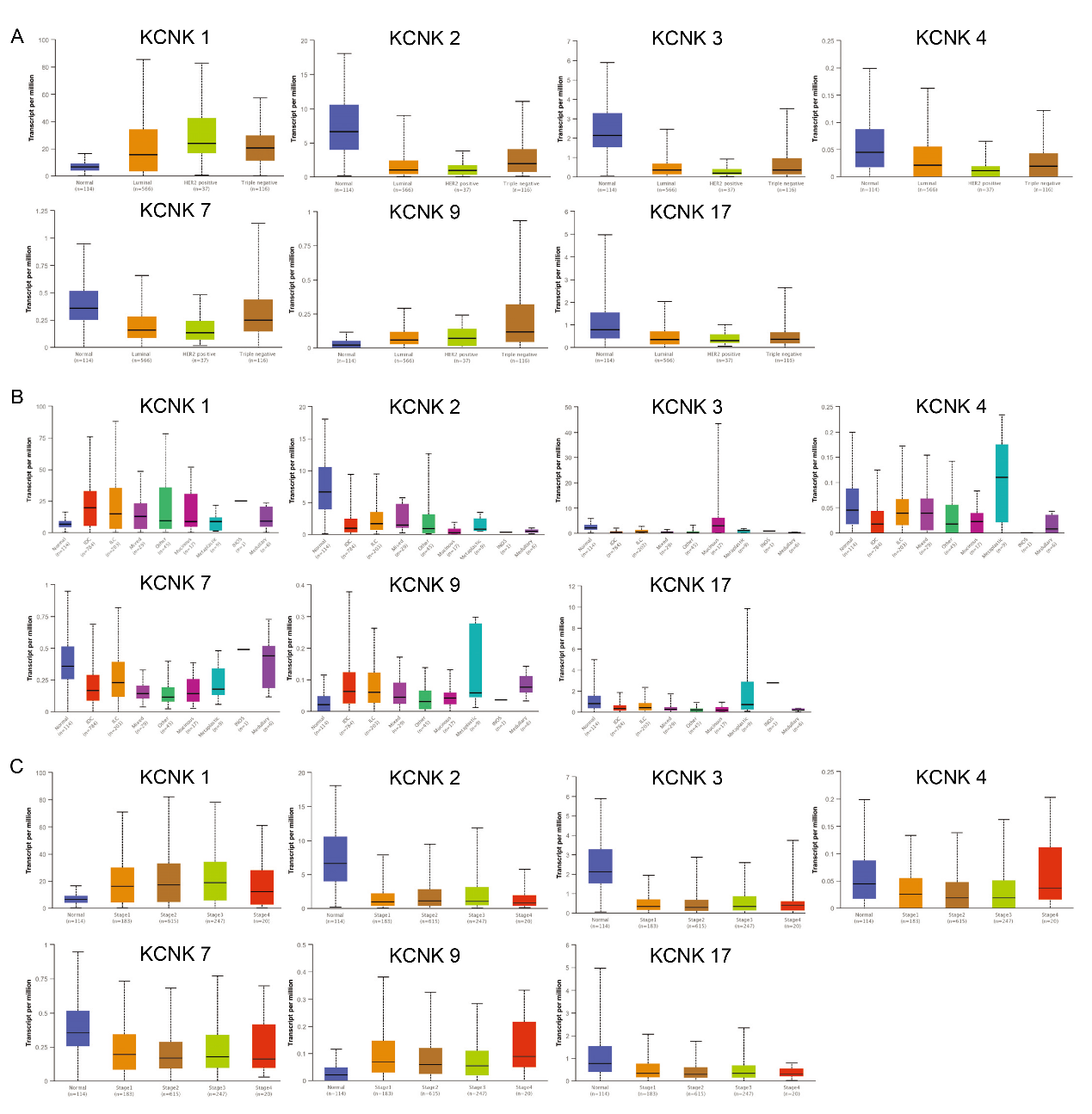
**Supplement Figure S2.** Boxplots of the relationship between the expression levels of each model gene and the breast cancer subtypes, histological subtypes, and pathologic stages.

**Supplement Figure S3.** Boxplots of the relationship between copy number variation of KCNKs and tumor microenvironment components.


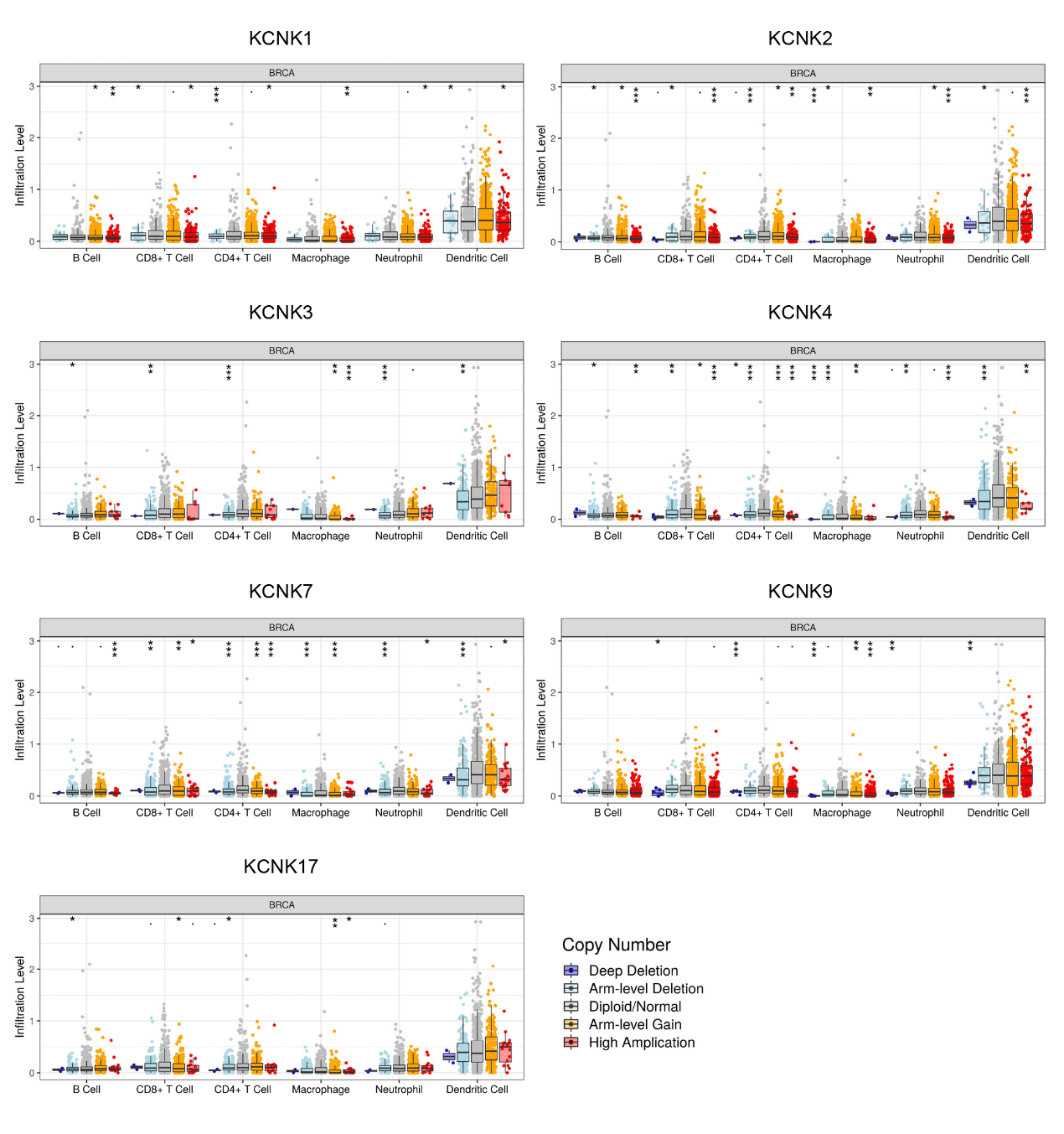

Supplement: Supplementary file 2 [file DataSheet1.DOCX]
